# Supplementary material for: Phenotyping of lymphoproliferative tumours generated in xenografts of non-small cell lung cancer
Source: Front Oncol. 2023 Jun 5;13:1156743. doi: 10.3389/fonc.2023.1156743 (PMC10277614; doi:10.3389/fonc.2023.1156743)

CRUK0701_R1_P0

IHC outcome: Lymphoproliferation.   Patient diagnosis: Squamous cell carcinoma.

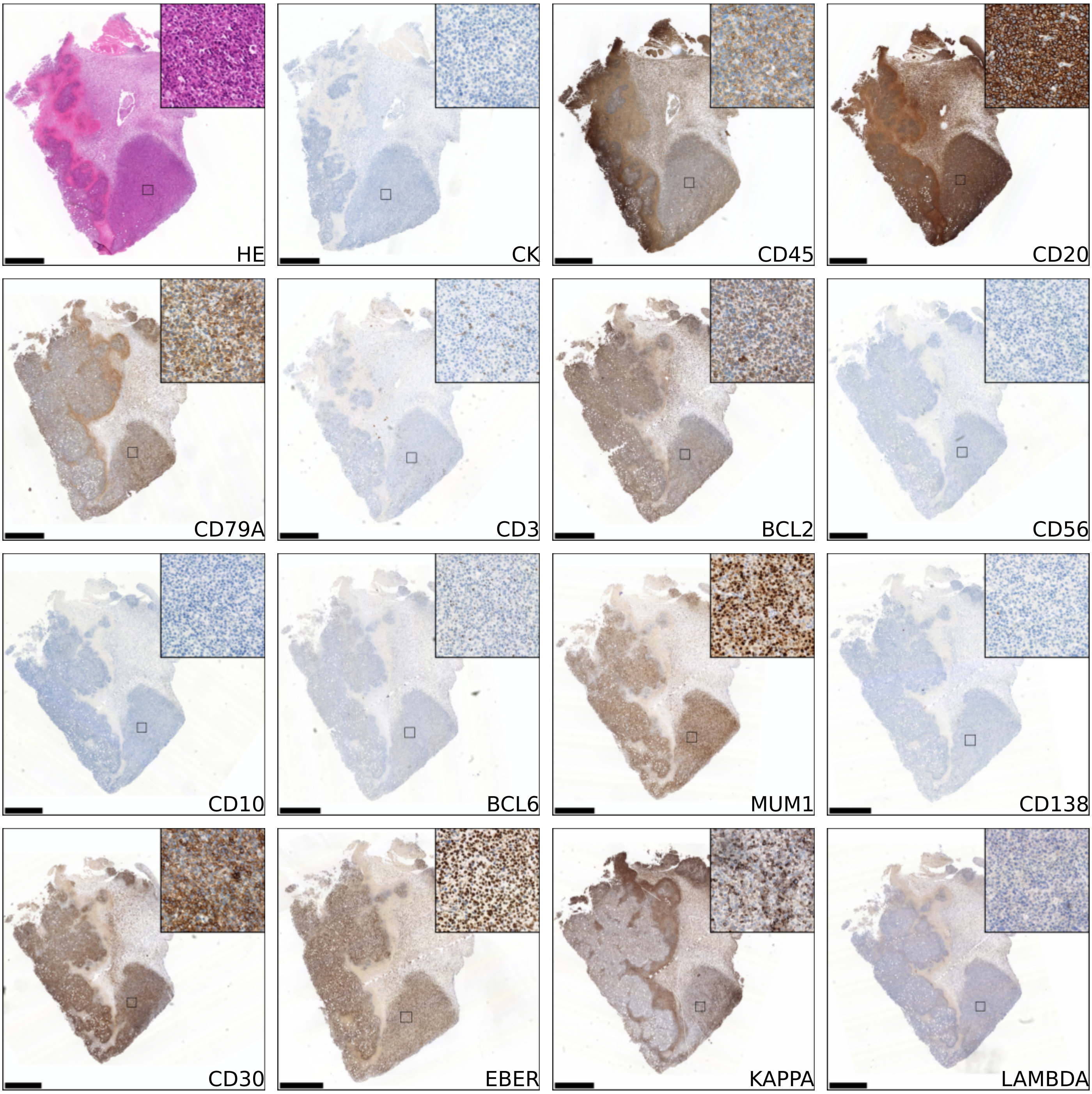

CRUK0704_T1-IR_P0

IHC outcome: Lymphoproliferation. Patient diagnosis: Invasive adenocarcinoma.

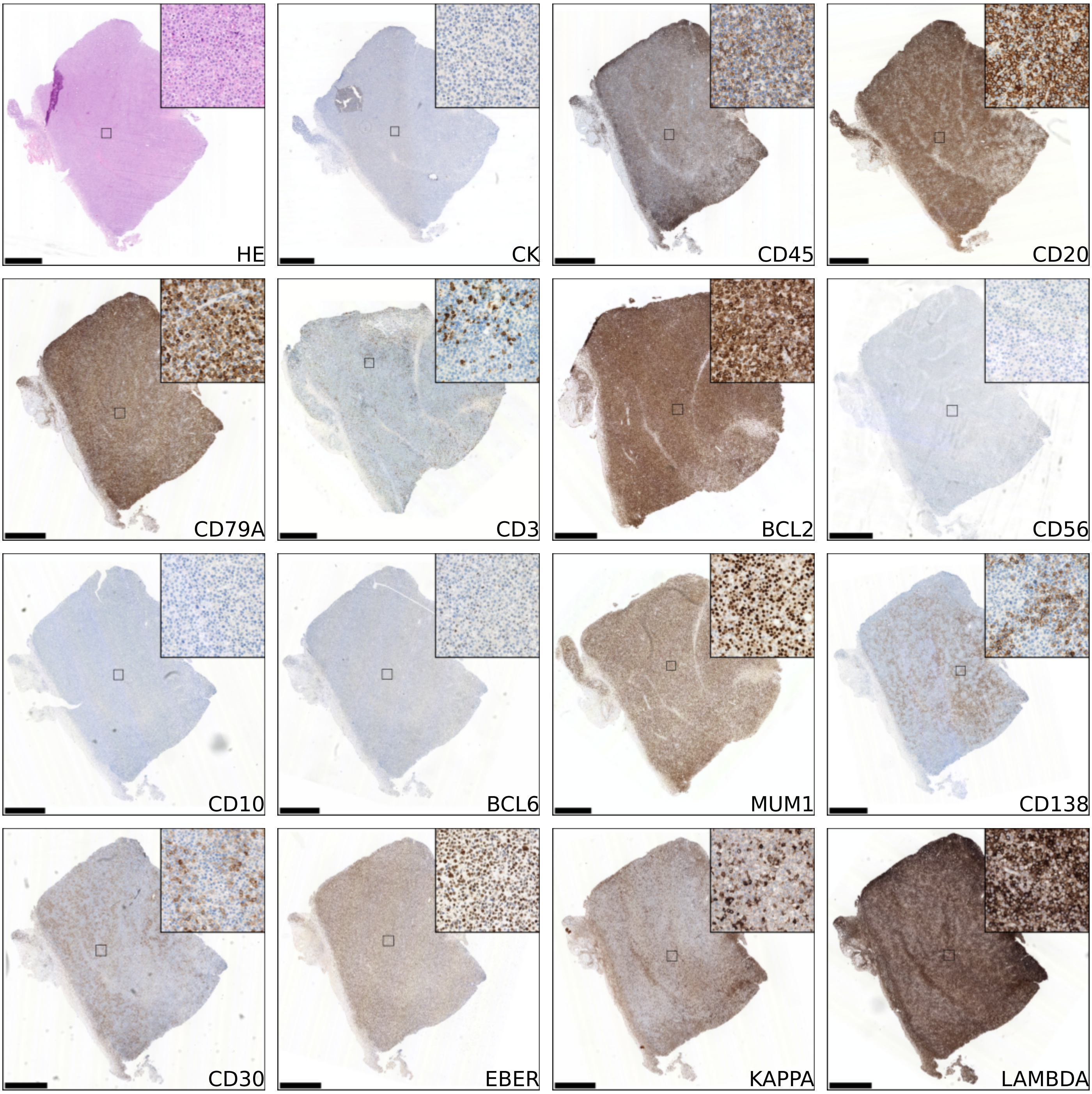

CRUK0731_IR_P0

IHC outcome: Lymphoproliferation.   Patient diagnosis: Squamous cell carcinoma.

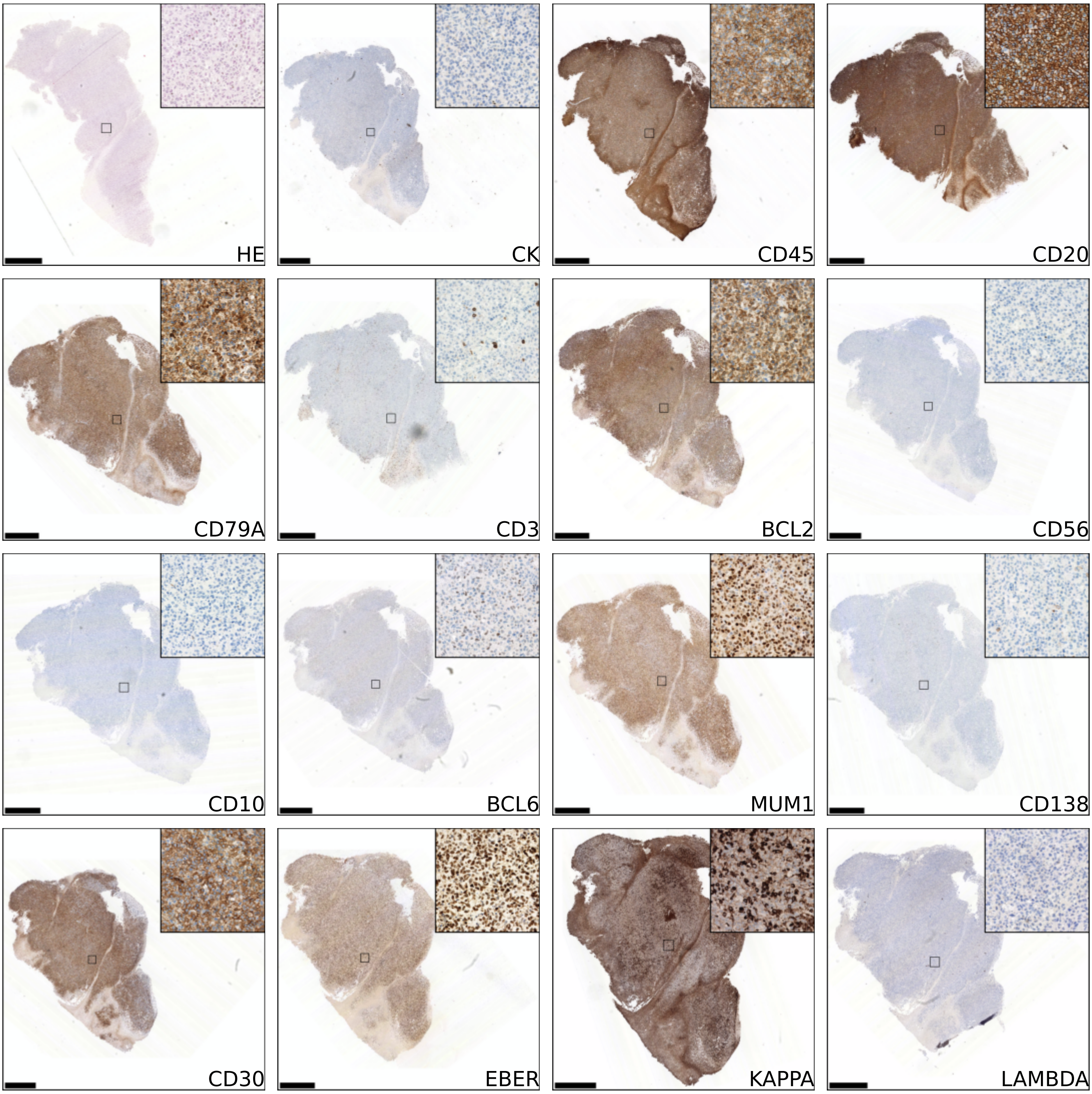

CRUK0733_R5_P0

IHC outcome: Lymphoproliferation.   Patient diagnosis: Invasive adenocarcinoma.

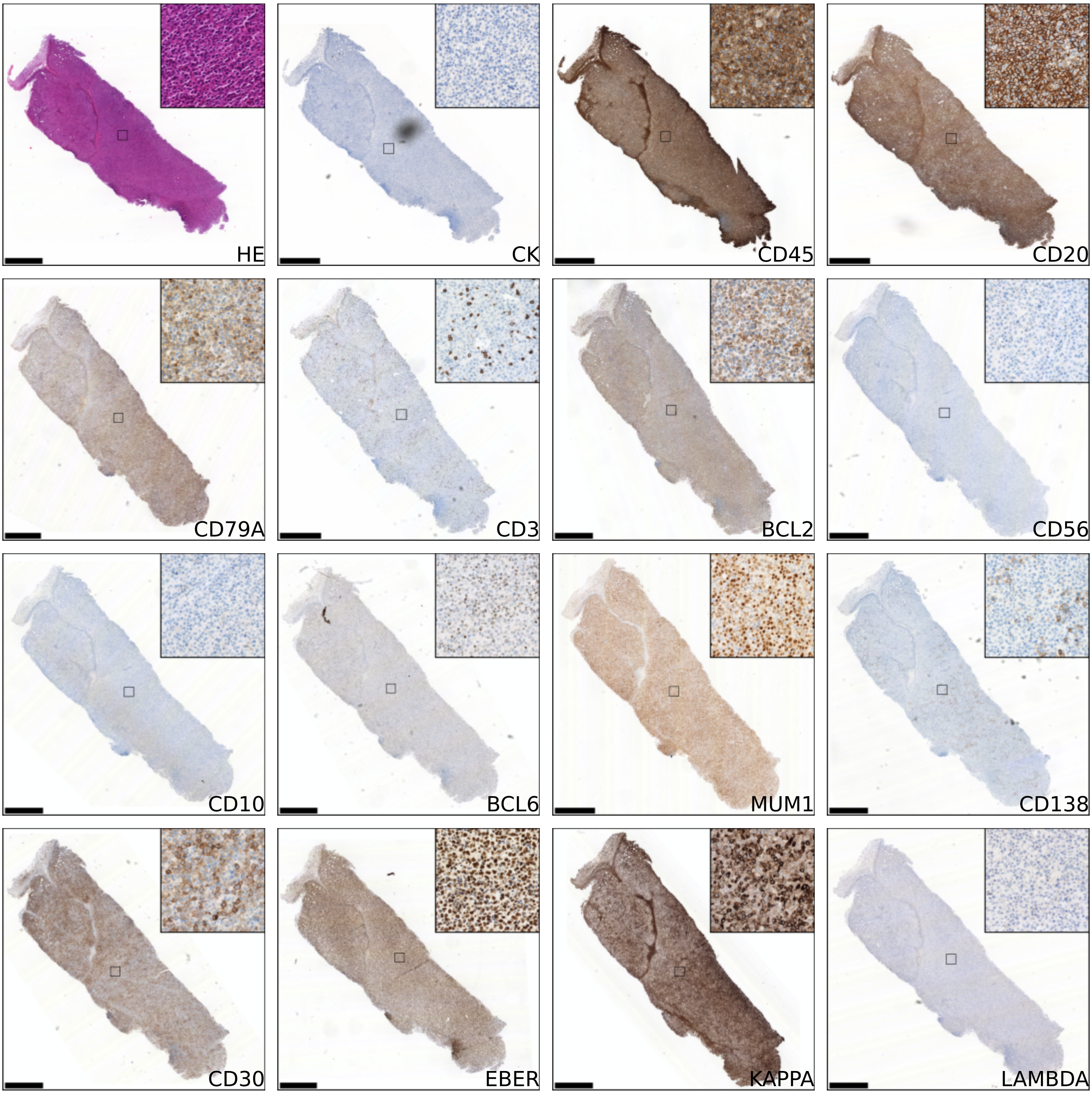

Created with PATHOverview. github.com/EpiCENTR-Lab/PATHOverview

CRUK0764_IR_P0

IHC outcome: Lymphoproliferation. Patient diagnosis: Invasive adenocarcinoma.

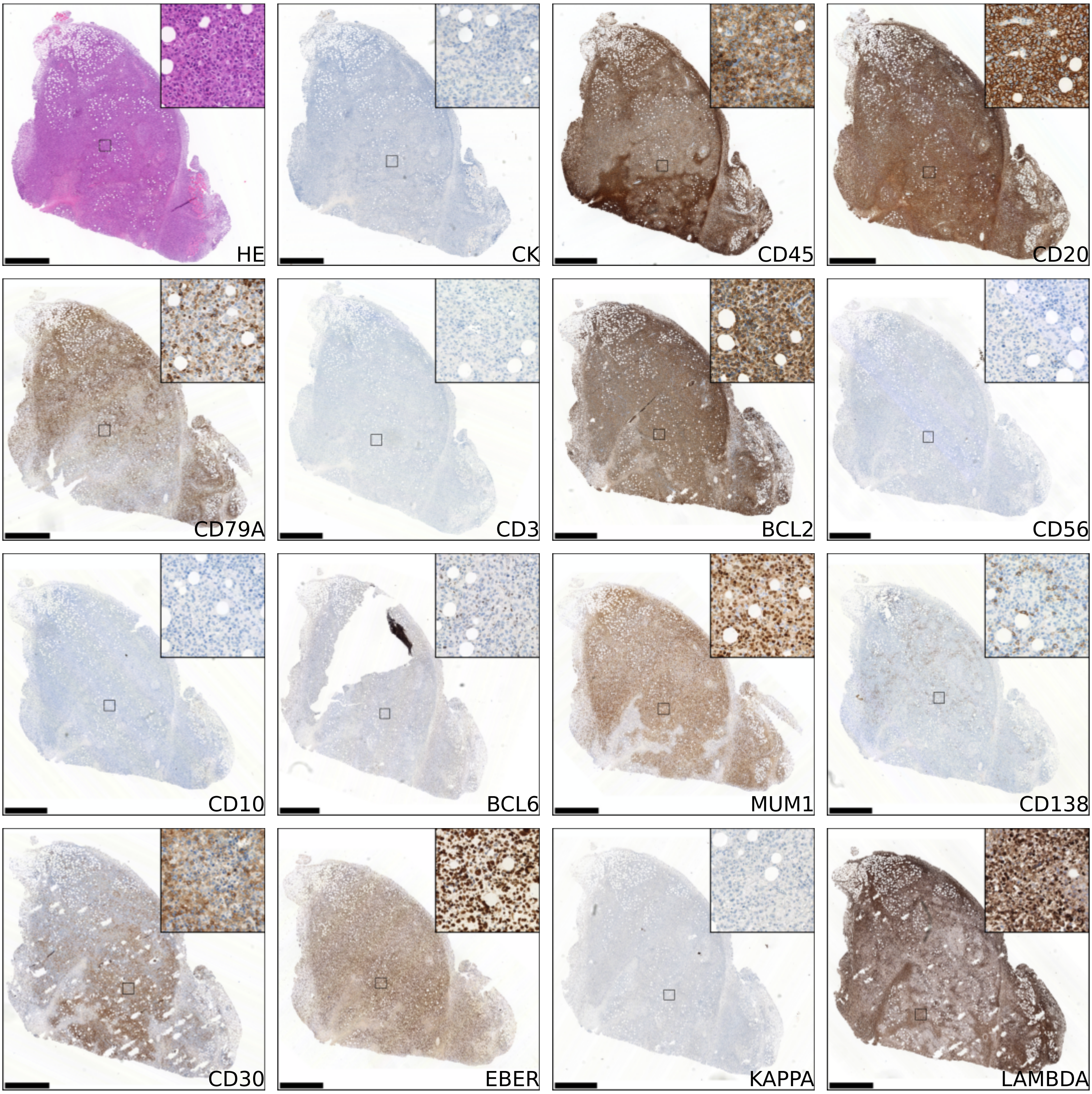

Created with PATHOverview. github.com/EpiCENTR-Lab/PATHOverview

CRUK0795_IR_P0

IHC outcome: Lymphoproliferation.   Patient diagnosis: Invasive adenocarcinoma.

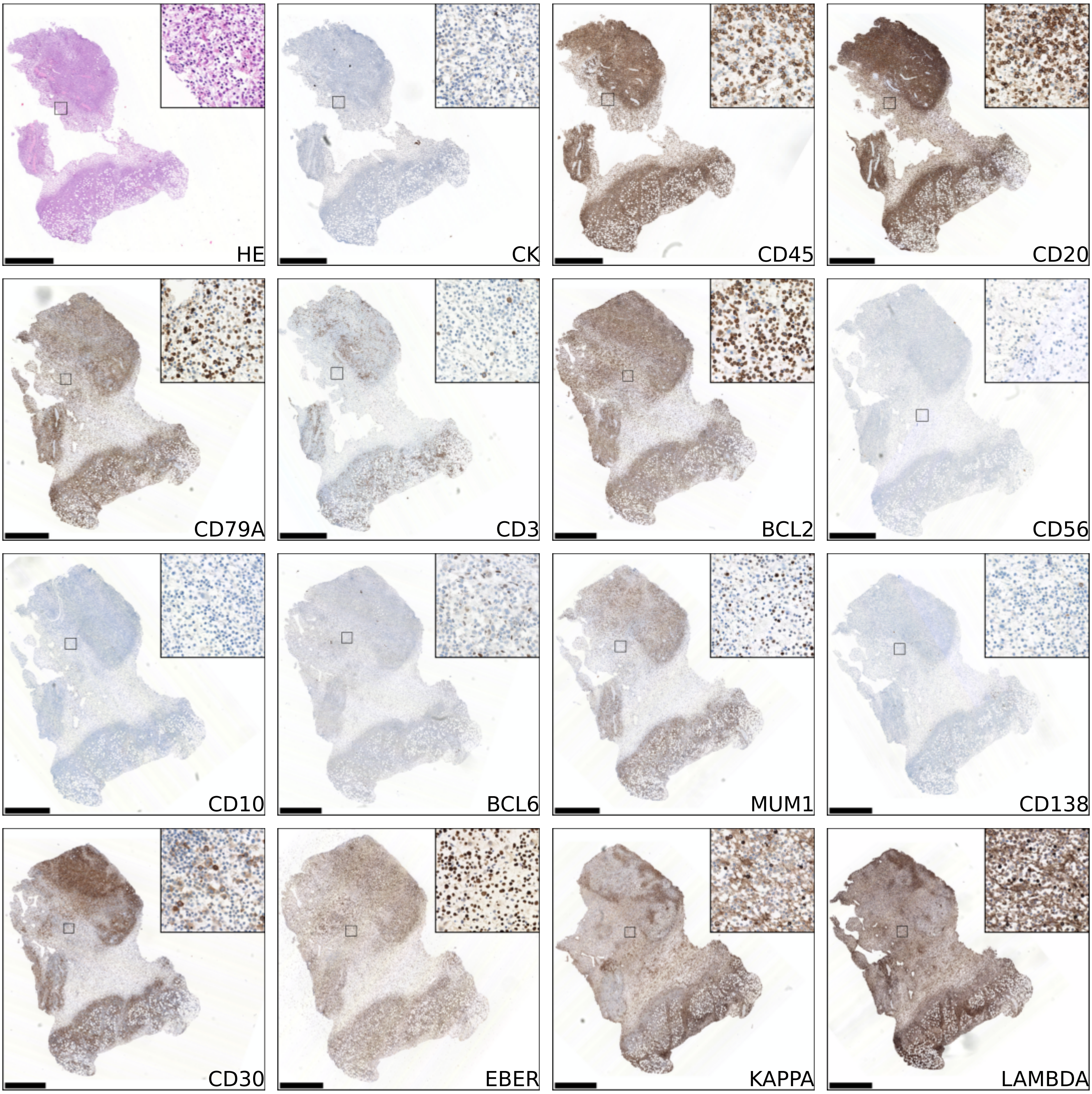

CRUK0810_R4_P0

IHC outcome: Lymphoproliferation.   Patient diagnosis: Invasive adenocarcinoma.

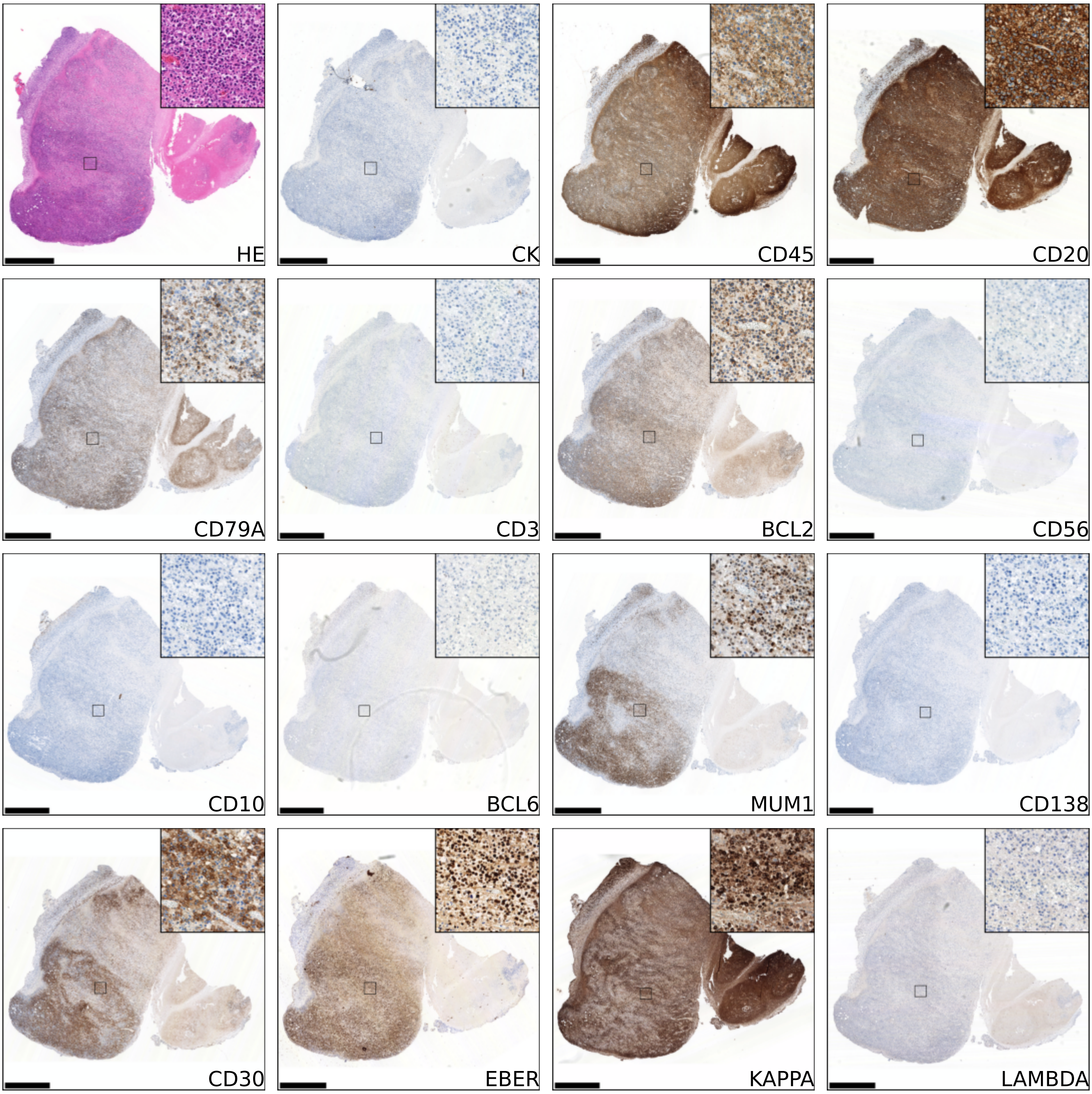

Created with PATHOverview. github.com/EpiCENTR-Lab/PATHOverview

CRUK0810_R8_P0

IHC outcome: Lymphoproliferation. Patient diagnosis: Invasive adenocarcinoma.

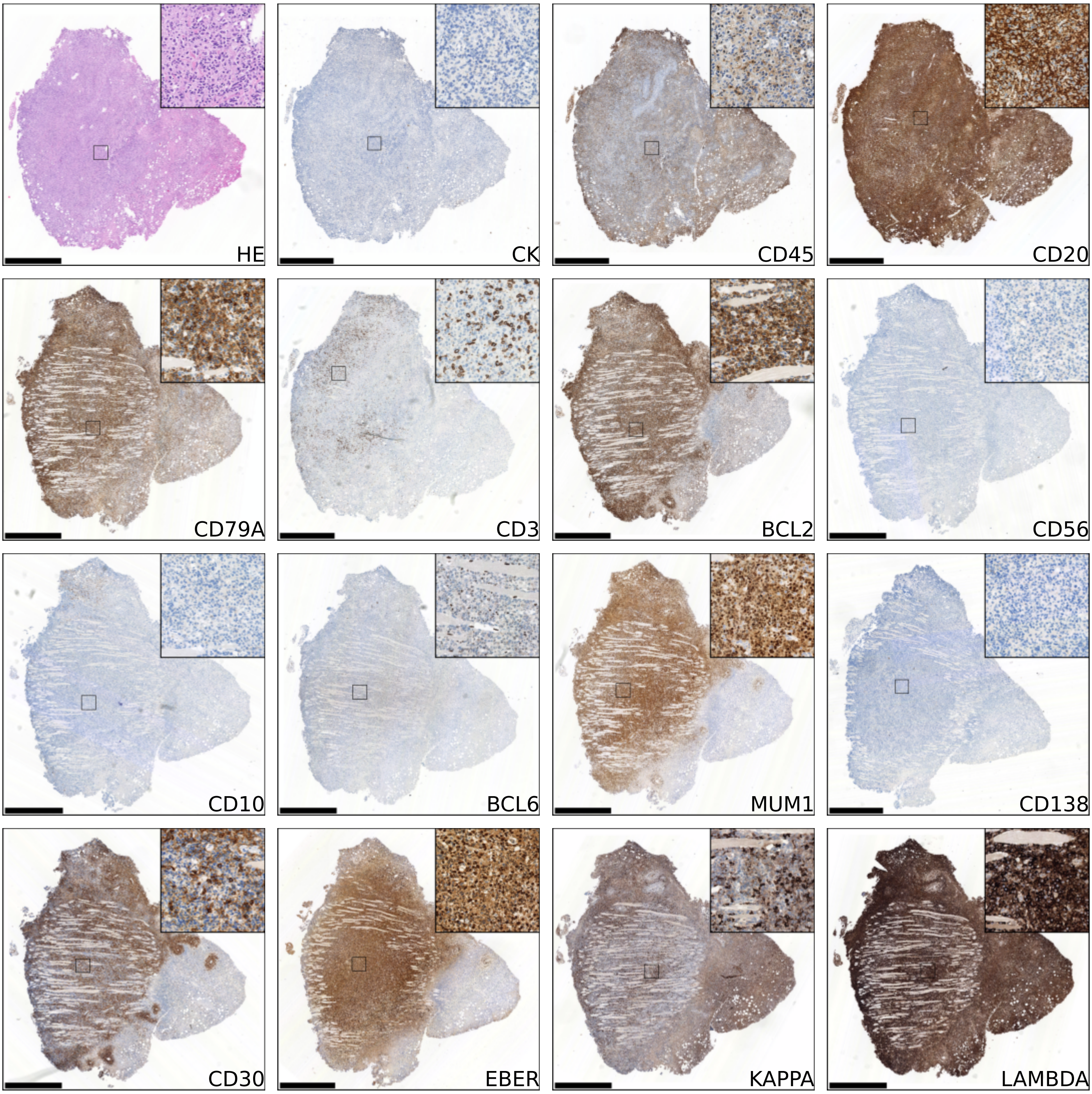

CRUK0825_R7_P0

IHC outcome: Lymphoproliferation.   Patient diagnosis: Invasive adenocarcinoma.

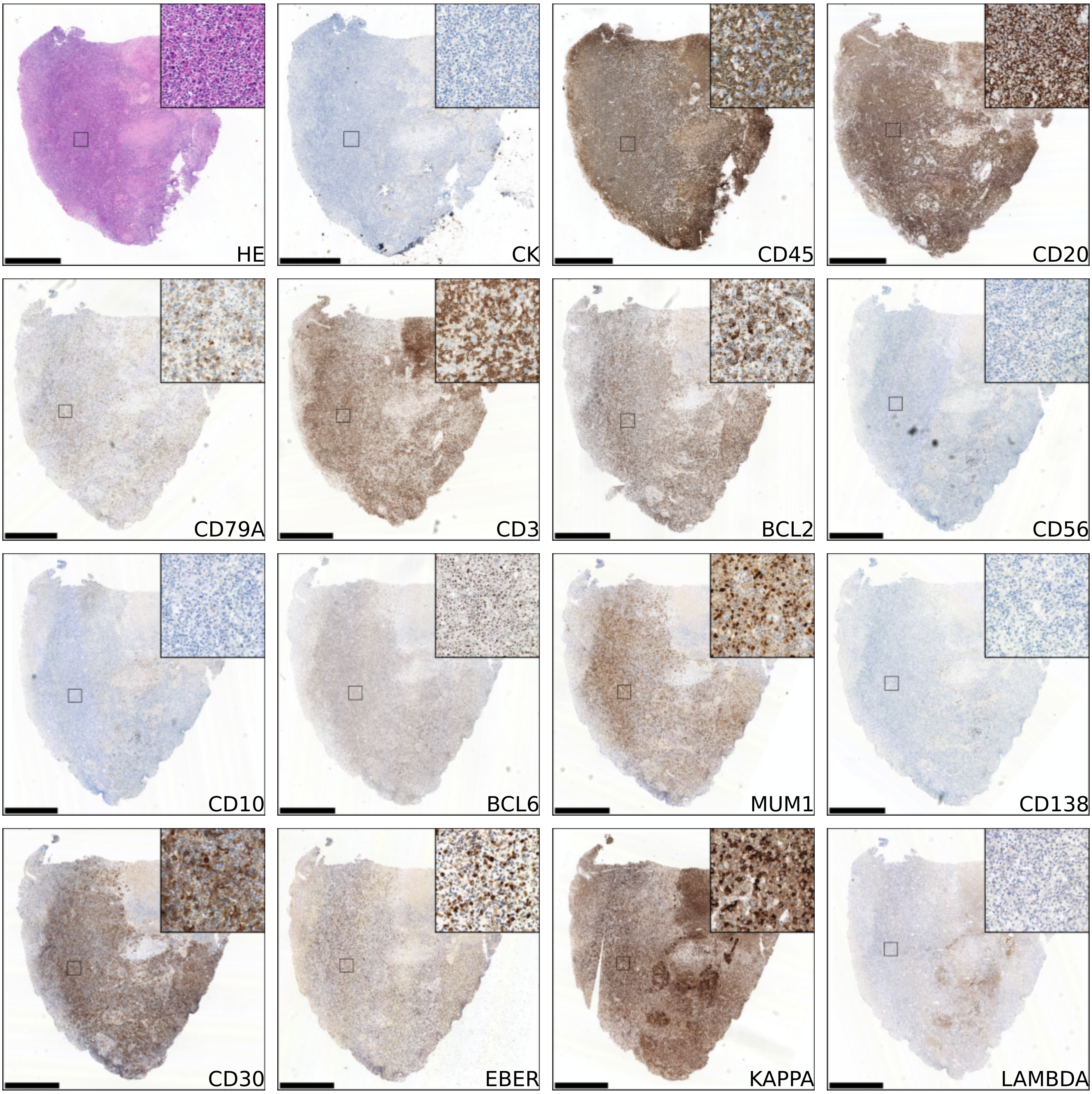

CRUK0830_R4_P0

IHC outcome: Lymphoproliferation. Patient diagnosis: Other.

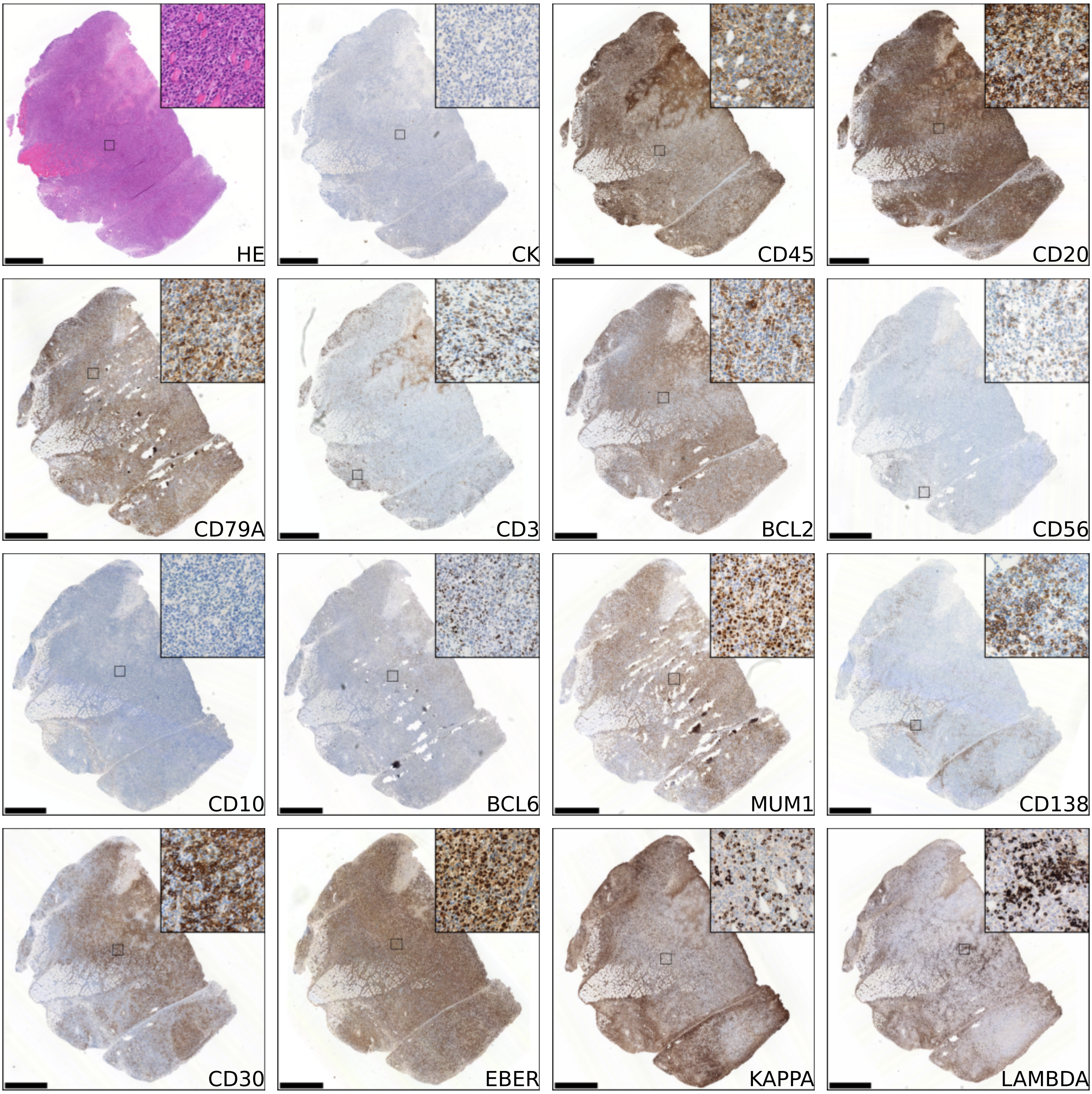

CRUK0934_R6_P0

IHC outcome: Lymphoproliferation.   Patient diagnosis: Squamous cell carcinoma.

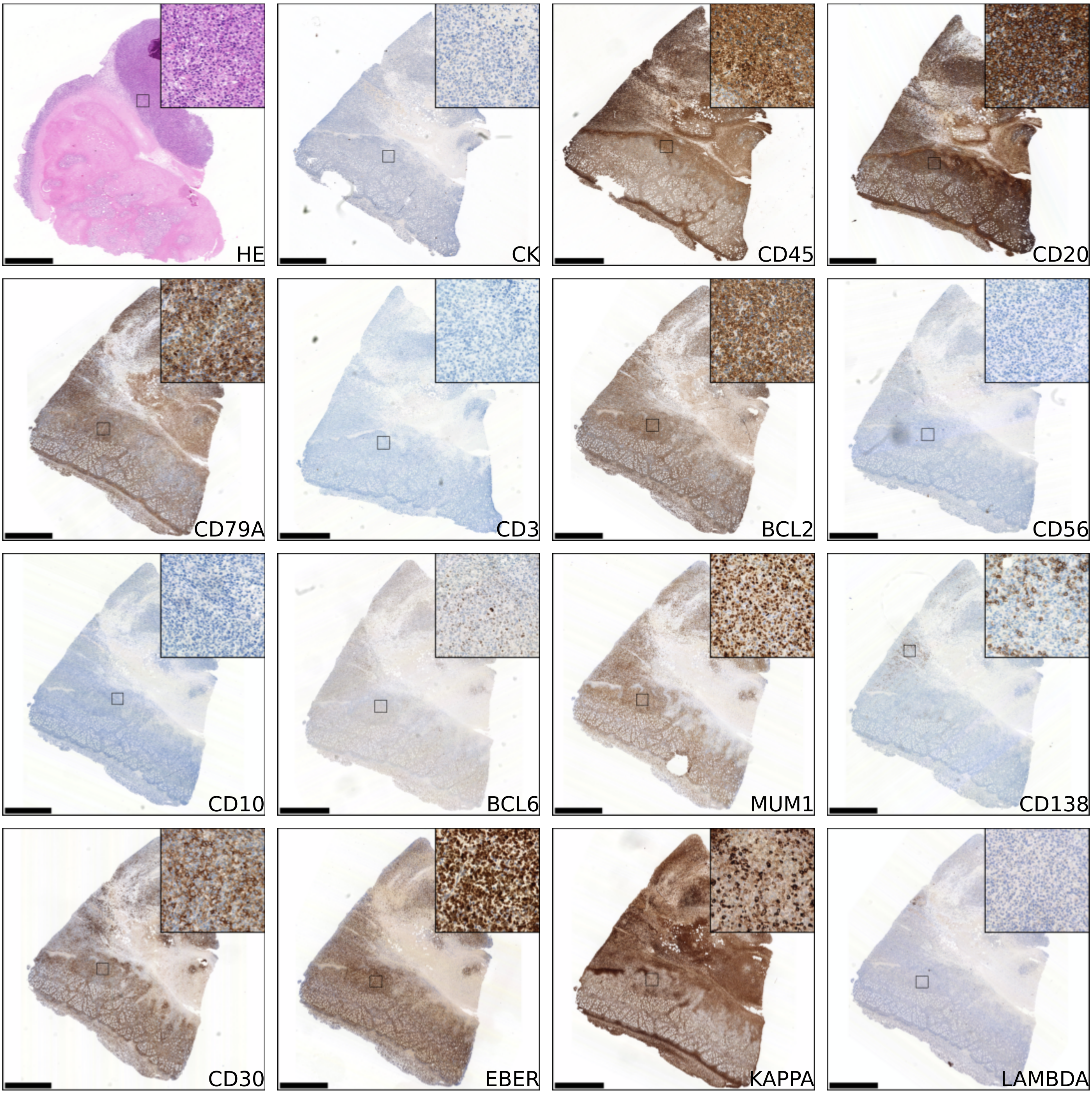

CRUK0941_R1_P0
IHC outcome: Lymphoproliferation.   Patient diagnosis: Invasive adenocarcinoma.

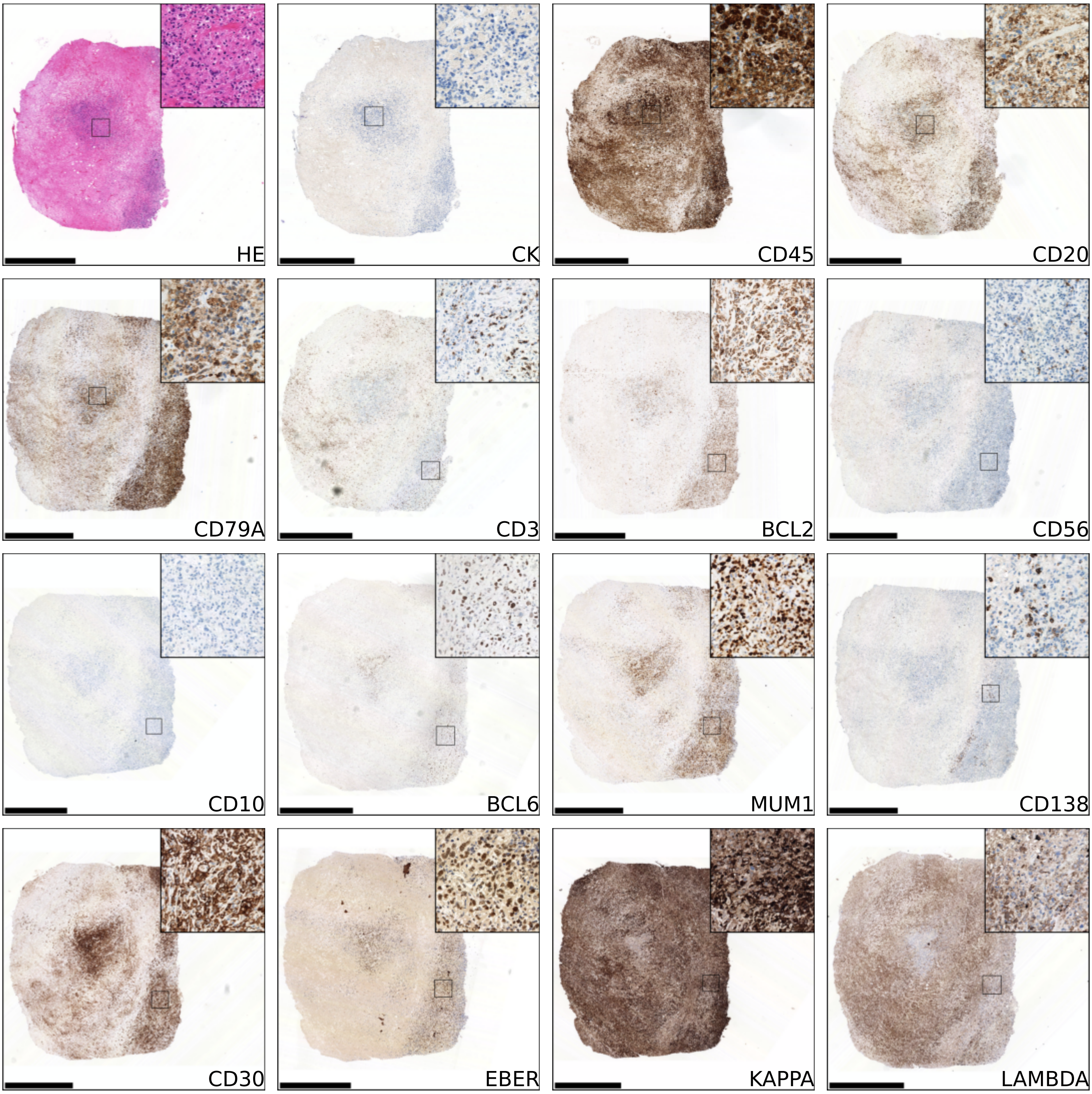

CRUK0949_R6_P0

IHC outcome: Lymphoproliferation. Patient diagnosis: Squamous cell carcinoma.

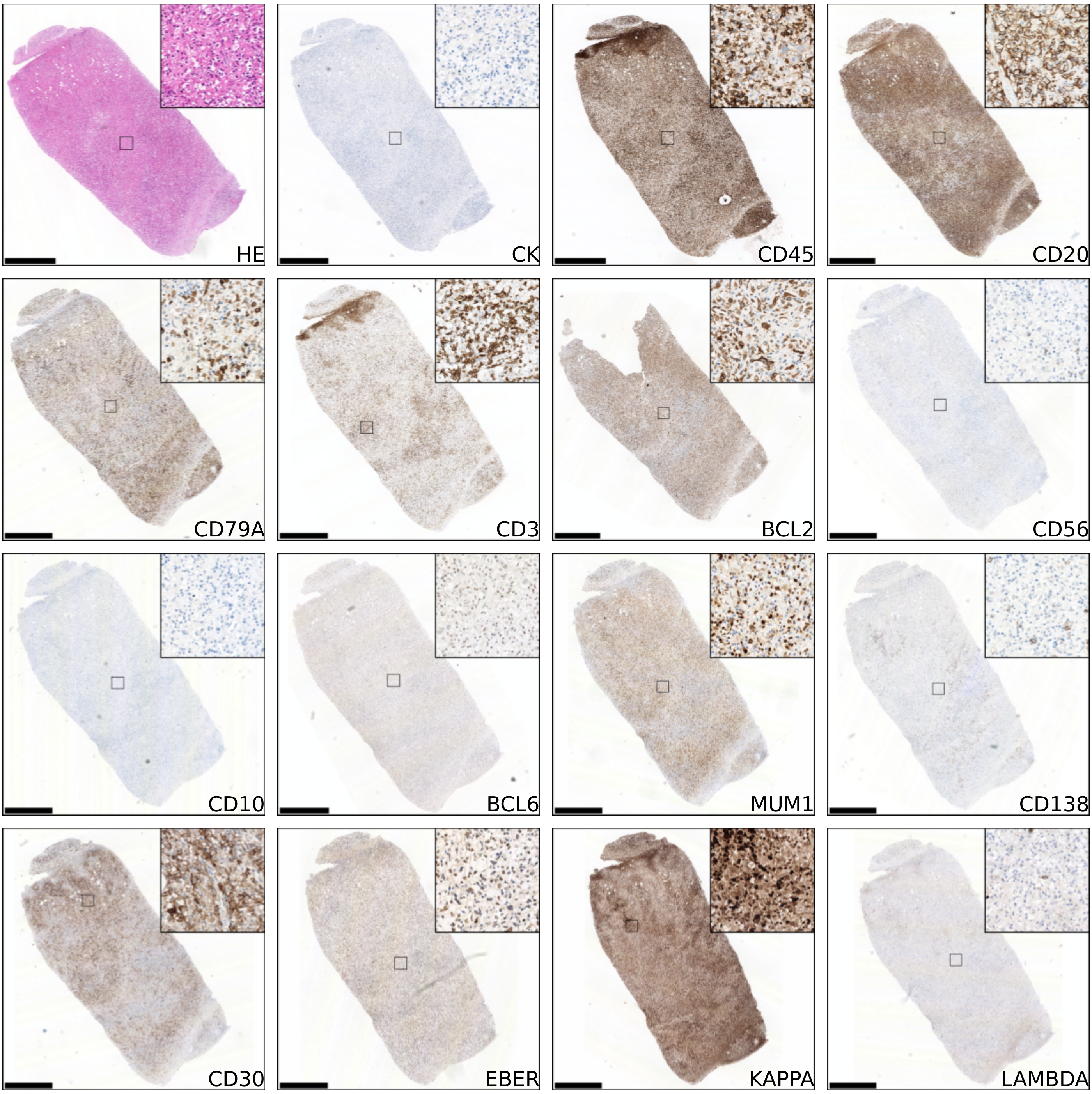

Created with PATHOverview. github.com/EpiCENTR-Lab/PATHOverview

CRUK1054_R2_P0
IHC outcome: Lymphoproliferation.   Patient diagnosis: Squamous cell carcinoma.

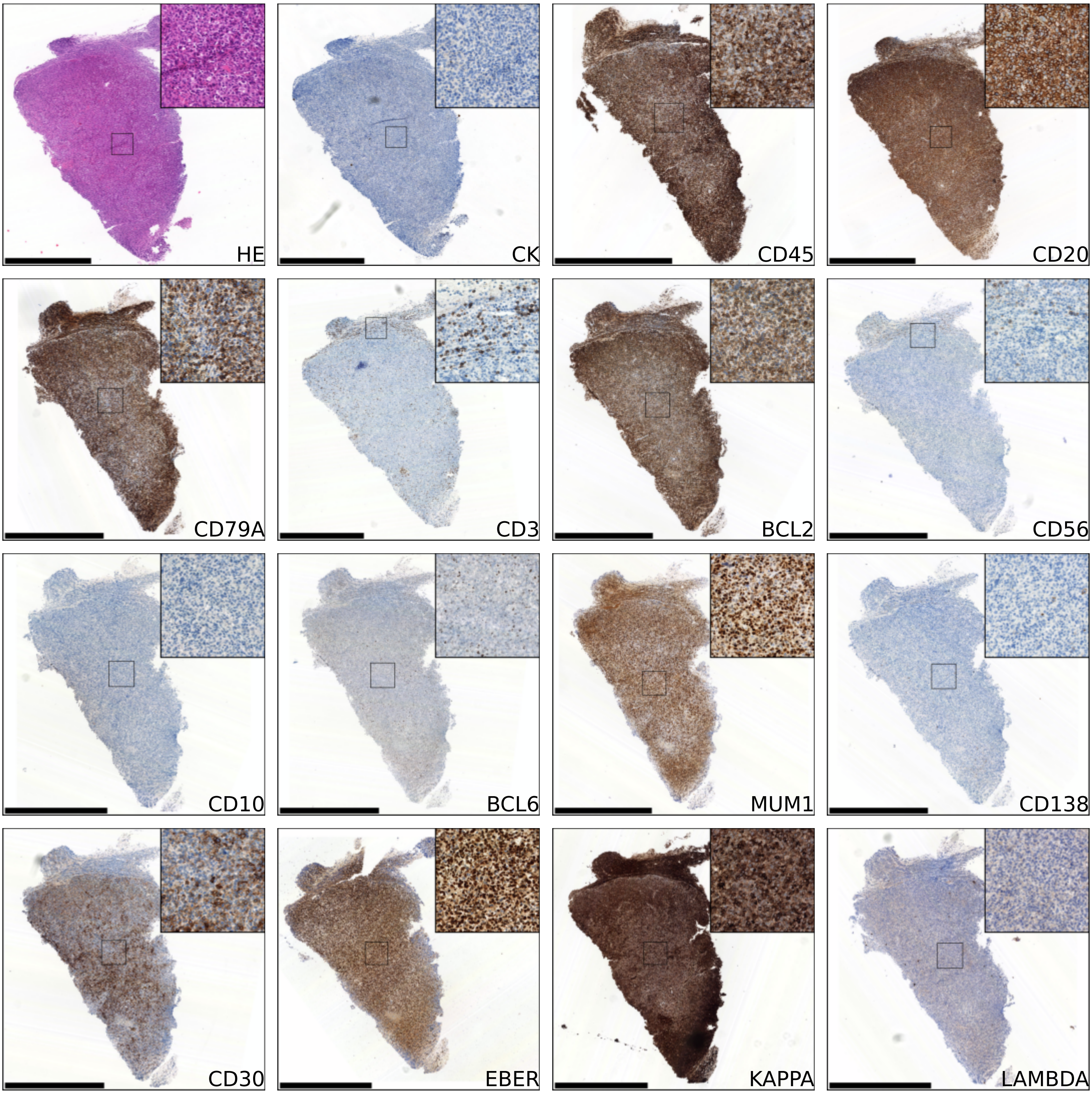

CRUK1054_R5_P0

IHC outcome: Lymphoproliferation.   Patient diagnosis: Squamous cell carcinoma.

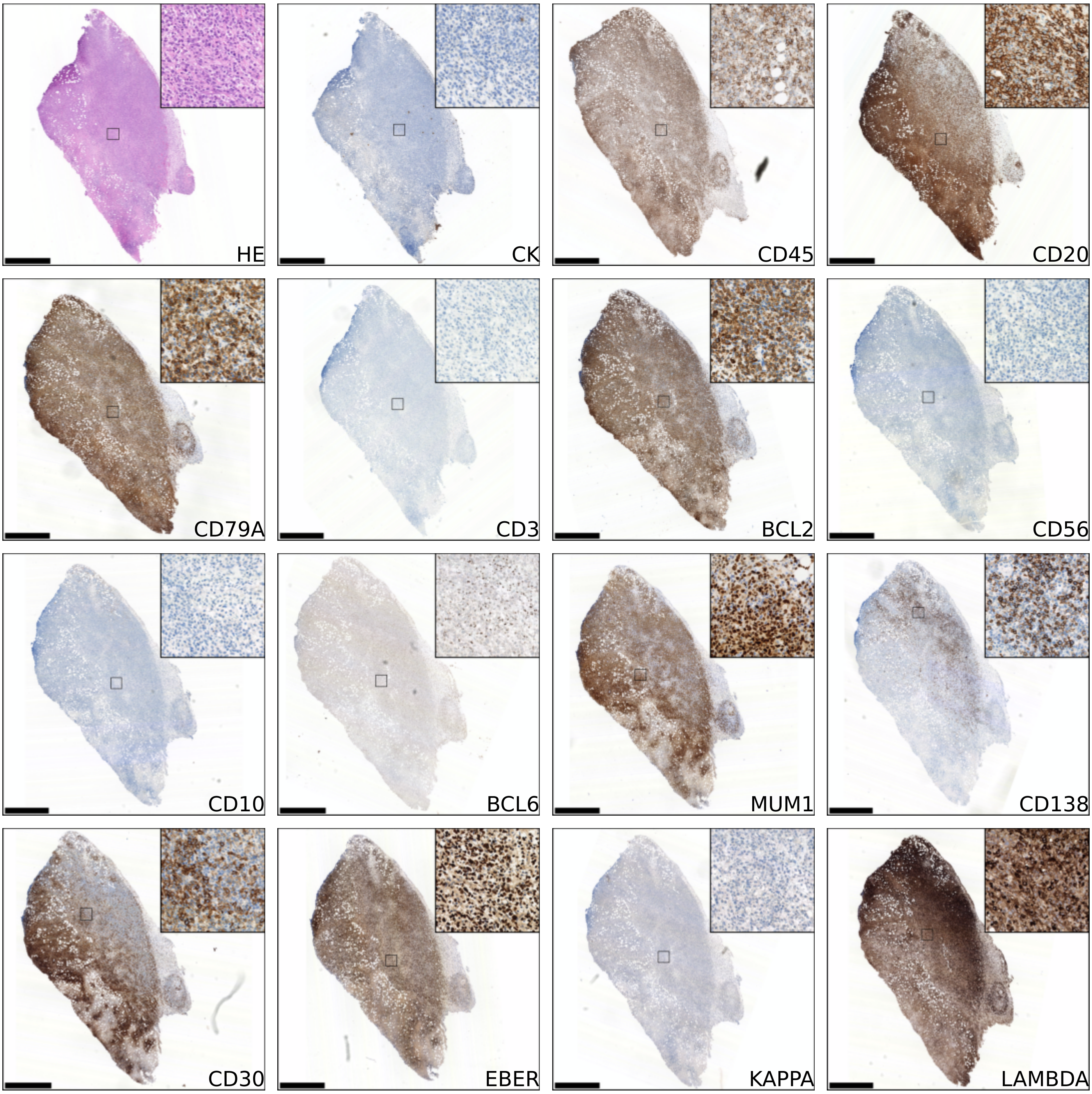

Supplement: Supplementary file 1 [file DataSheet_1.pdf]
